# The Molecular Mechanism by Which miR-129a-3p Targets the TLR4/NF-κB Signaling Pathway to Regulate Inflammatory Damage in 3D4/21 Cells Infected with *Glaesserella parasuis*

**DOI:** 10.3390/ani15101355

**Published:** 2025-05-08

**Authors:** Zhongbo Guo, Yuanyuan Zhou, Na Li, Aobo Shen, Yongchao Jia, Ronglan Yin, Junjie Yang, Jing Yuan, Ronghuan Yin

**Affiliations:** 1 Key Laboratory of Livestock Infectious Diseases, Ministry of Education, and Key Laboratory of Ruminant Infectious Disease Prevention and Control (East), Ministry of Agriculture and Rural Affairs, College of Animal Science and Veterinary Medicine, Shenyang Agricultural University, 120 Dongling Road, Shenyang 110866, China; 15048055087@163.com (Z.G.); zhouyy26@syau.edu.cn (Y.Z.); lina001028@163.com (N.L.); 15642032160@163.com (A.S.); jiayongchao0320@163.com (Y.J.); yangjunjie8051@163.com (J.Y.); 2 Research Academy of Animal Husbandry and Veterinary Medicine Sciences of Jilin Province, Changchun 130062, China; yinronglan@163.com

**Keywords:** *Glaesserella parasuis*, inflammatory damage, microRNAs, ssc-miR-129a-3p, NF-κB pathway

## Abstract

*Glaesserella parasuis* (*G. parasuis*) is the primary pathogen responsible for Glässer’s disease and poses a significant threat to the global pig industry. MicroRNAs are a class of short, endogenous, single-stranded noncoding RNAs that play crucial roles in inflammation, apoptosis, proliferation, differentiation, and invasion in various organisms. This study analyzed the characteristics of porcine alveolar macrophage (PAM) cells infected with *G. parasuis* through the knockdown and overexpression of ssc-miR-129a-3p. We constructed a cellular model with ssc-miR-129a-3p knockdown invaded by *G. parasuis* strain XX0306, screening 160 differentially expressed genes via high-throughput sequencing. GO enrichment analysis revealed that 376 GO entries were enriched. KEGG enrichment analysis found that mRNA target genes were enriched in 17 cell signaling pathways, including G protein-coupled components, PPAR, and other signaling pathways that can mediate inflammatory pathways. By examining the expression of relevant inflammatory factors and signaling pathways, we elucidated the molecular mechanisms through which ssc-miR-129a-3p targets the TLR4/NF-κB signaling pathway to regulate inflammatory injury. This study establishes a foundation for further research into the role of miRNA in the pathogenesis of Glässer disease and is highly significant for the prevention and control of bacterial diseases within the pig industry.

## Simple Summary

miR-129a-3p plays an important role in the inflammatory response induced by *G. parasuis* infection. The TLR4/NF-κB signaling pathway is an important inflammatory signaling pathway. The results of this study showed that miR-129a-3p targeted the TLR4/NF-κB signaling pathway to regulate the inflammatory injury caused by *G. parasuis*-infected porcine alveolar macrophage cells, providing new insights into the role of miRNA in the pathogenic mechanism *G. parasuis*.

## 1. Introduction

*Glaesserella parasuis* (*G. parasuis*) is a significant pathogenic bacterium that causes Glässer’s disease, which in turn causes a systemic inflammatory response [1]. Sick pigs are mostly characterized by fibrous polyserositis, meningitis, and arthritis [1]. In severe cases, the disease can lead to death, resulting in considerable economic losses for the pig industry [2,3,4,5,6,7]. Previous studies have focused on virulence and epidemiology, but more recently, *G. parasuis* has been found to induce the production of several inflammatory factors, such as IL-6, IL-1β, and TNF-α. However, the molecular mechanisms underlying the interaction between *G. parasuis* and host cells remain largely unknown, complicating efforts for disease prevention and control [8,9].

MicroRNAs (miRNAs) are endogenous non-coding RNAs (ncRNAs) that play a crucial role in a variety of diseases [10,11,12]. These molecules have been implicated in the regulatory mechanisms of various diseases [13,14,15,16], whether as part of the host immune response to neutralize infections or as molecular strategies employed by bacteria to hijack host pathways for their own benefit [17]. For instance, extracellular vesicle (EV) transporter miRNAs exhibit significant systemic involvement in polytrauma [18]. Furthermore, miRNAs are involved in all known processes associated with cancer, including proliferation, survival, metastasis, and apoptosis [19,20,21,22,23]. Notably, the MS-CeO2-miR129 complex has been shown to alleviate inflammation in RISI wounds while promoting granulation tissue formation, angiogenesis, and collagen deposition, thus facilitating wound closure [24]. Additionally, miR129-2 has demonstrated the ability to inhibit tumorigenesis [25]. MiR-129-5p is also being explored as a potential biomarker for Alzheimer’s disease pathology and cognitive decline [26]. Moreover, miR-129 protects mice from sepsis-induced acute lung injury (ALI) by attenuating lung inflammation and apoptosis through the regulation of the TAK1/NF-κB signaling pathway [27].

In terms of bacterial infections, research has found that exosomal-miR-129-2-3p derived from Fusobacterium nucleatum-infected intestinal epithelial cells promotes experimental colitis by regulating the TIMELESS-mediated cellular senescence pathway [28]. Exosomal miR-155-5p drives widespread macrophage M1 polarization in hypervirulent Klebsiella pneumoniae-induced acute lung injury via the MSK1/p38-MAPK axis [29]. Has-miR-30c-1-3p inhibits macrophage autophagy and promotes Mycobacterium tuberculosis survival by targeting ATG4B and ATG9B [30]. There are also studies indicating that some Helicobacter pylori-related miRNAs are expected to become therapeutic tools and biomarkers for the prevention, diagnosis, and prognosis of gastric cancer (GC) [31]. Similarly, exosomal miRNAs are also biomarkers in infection with Mycobacterium tuberculosis, and miRNAs present potential as therapeutic targets in host-directed therapy (HDT) techniques for TB infection [32,33]. MicroRNA-223 dampens pulmonary inflammation during pneumococcal pneumonia [34].

Although the aforementioned studies have demonstrated that miR-129 plays a significant role in various diseases, research on the miRNA regulatory network in bacterial infections, particularly in *G. parasuis*, remains relatively scarce. There is a lack of systematic analysis regarding the regulatory functions of miRNAs, and it has not been conclusively verified in previous studies whether miR-129a-3p is involved in pathways related to bacterial infections, leading to its function being unresolved. In response to the current research status, we have, for the first time, revealed the expression characteristics and functions of miR-129a-3p in *G. parasuis*. Through RT-qPCR, we discovered that the expression of miR-129a-3p is significantly upregulated in the lung tissues of pigs infected with *G. parasuis*, suggesting its potential involvement in the host inflammatory response. Through interference overexpression functional assays, it was demonstrated that it plays a regulatory role in inflammatory responses. Subsequently, transcriptome sequencing analysis was conducted to identify inflammatory signaling pathways, and bioinformatics analysis was used to pinpoint the target sites. Ultimately, it was elucidated that miR-129a-3p regulates the specific mechanism of inflammatory injury in PAM cells infected with *Glaesserella parasuis* through the TLR4/NF-κB signaling pathway. It is hoped that this work can guide the application of miR-129a-3p and its targets in future treatments, laying the foundation for research on the prevention of Glässer’s disease.

## 2. Materials and Methods

### 2.1. Ethics Statement

The Ethical Review Committee and Laboratory Animal Welfare Committee of Shenyang Agricultural University, China, approved all experimental protocols (No. 201806014).

### 2.2. Bacterial and Cell Culture Conditions

PAM cells (Xinrun Biologicals, Wuxi, China) were cultured in 1640 medium (Hyclone, Beijing, China) supplemented with 10% fetal bovine serum (FBS, Lonsera, Uruguay), 100 U/mL penicillin (Solarbio, Beijing, China), and 100 mg/mL streptomycin (Solarbio, Beijing, China) at 37 °C in a humidified atmosphere containing 5% CO_2_. The highly virulent *G. parasuis* clinical isolate XX0306 (serotype 5) was maintained in our laboratory and cultured on tryptic soy agar medium (TSA, Solarbio, Beijing, China) without antibiotics, supplemented with 0.2 mg/mL nicotinamide adenine dinucleotide (NAD, Solarbio) and 10% fetal bovine serum (Dingguo, Beijing, China). After overnight incubation at 37 °C, the bacteria were harvested by centrifugation at 5000× *g* for 5 min. Then, the precipitates were washed three times with PBS and resuspended in a fresh 1640 medium for the in vitro infection of PAM cells.

### 2.3. Animals and Sample Collection

The piglets utilized in this study were sourced from the Xinmin Shuoyang Breeding Specialized Cooperative (Xinmin City, Liaoning Province, China) and were fed and reared in a consistent environment following standard feeding management practices. The piglets were divided into two groups: a blank control group (6 pigs, 30 days old) and a bacteria attack group (6 pigs, 30 days old). Each group was housed and fed separately, and three individuals from each group were randomly selected for sample collection.

Piglet heart, liver, spleen, lung, and kidney tissues, thoroughly washed with phosphate-buffered saline (Servicebio, Wuhan, China), were collected and stored in liquid nitrogen for the subsequent analysis of tissue data.

### 2.4. Cell Transfection and Processing

When the 12-well plate reached about 50–60% of the cell volume, ssc-miR-129a-3p mimics, mimic NC, ssc-miR-129a-3p inhibitor, inhibitor NC, siTLR4, and NC were transfected using GP-transfect-Mate reagent (Gemma, Shanghai, China) (Table 1). Transfection concentrations were taken from the instruction manual.

### 2.5. RNA Extraction and Real-Time Fluorescence Quantitative PCR

Total RNA was extracted using the TRIzol method. MiRNA and total RNA were reverse-transcribed using the Vazyme kit (Vazyme, Nanjing, China) according to the instructions. For each sample, 1 μg of RNA was used for reverse transcription. The expression of relevant genes was detected separately by RT-qPCR. PCR mixtures were as follows: 5 μL of SYBR; 10 μmol/L of each of the upstream and downstream primers; 0.2 μL of each primer; 1 μL of the template; and water without RNase. Finally, relative quantification was performed using the 2^−∆∆Ct^ method. Detailed information on the primer sequences is shown in Table 2, Table 3 and Table 4.

### 2.6. Transmission Electron Microscopy (TEM)

Transmission electron microscopy (TEM) was employed to observe and analyze the impact of the miR-129a-3p inhibitor on the lesions in *G. parasuis*-infected PAM cells. The corresponding treated cells were washed twice with sterile PBS and subjected to overnight fixation in 2.5% glutaraldehyde solution. Subsequently, the samples were further fixed with osmium tetroxide and embedded in epoxy resin. Finally, the sections were stained, observed, and photographed using TEM (JEOL Ltd., Tokyo, Japan).

### 2.7. RNA-Seq Data Analysis

PAM cells with miR-129a-3p inhibitor or inhibitor NC, infected by *G. parasuis*, were submitted to LC-Bio Technologies Co., Ltd. (Hangzhou, China) for transcriptome sequencing. FastQC was employed to assess the quality of the raw sequencing data, and clean reads were aligned with the porcine reference genome using Hisat2. Notably, high-quality clean read lengths and unique sequence information were obtained. Transcripts were reconstructed using StringTie, and expression levels were calculated for all genes in each sample. Finally, significant differentially expressed genes (DEGs) were identified using DESeq2 (*p* < 0.05, |log_2_Foldchange| ≥ 1).

### 2.8. Dual-Luciferase Assay

The target genes were predicted online using Microbiotics miRanda, and TLR4 and NLRP3 sequences were obtained from the NCBI database (https://www.ncbi.nlm.nih.gov (accessed on 20 February 2025)). Cells were co-transfected with miR-129a-3p mimics, and the recombinant vectors PmirGLO-TLR4 WT, PmirGLO-TLR4 MUT, PmirGLO-NLRP3 WT, and PmirGLO-NLRP3 MUT. Following transfection, cells were completely lysed by the addition of cell lysate and centrifuged at 12,000× *g* for 3–5 min. The supernatant was then collected for the assay. Firefly luciferase and sea kidney luciferase activities were measured according to the instructions provided in the Dual-Luciferase Reporter Gene Assay Kit, and the ratio of the two activities was calculated to indicate relative luciferase activity. Plasmid sequence information is presented in Table 5.

### 2.9. Western Blot Analysis

Details of the antibodies used in this study are presented in Table 6. Proteins were extracted using RIPA lysis buffer (Solarbio, Beijing, China) supplemented with 1 mM PMSF (PMSF, Solarbio, Beijing, China), and protein concentrations were determined using the BCA assay kit (Beyotime, Shanghai, China). To separate the protein samples according to the size of the target proteins, 12% sodium dodecyl sulfate–polyacrylamide gel electrophoresis was employed, with 20 μg of protein loaded per well. Following electrophoresis, the separated proteins were transferred to a nitrocellulose (NC) membrane, which was subsequently blocked with 5% skimmed milk prepared in TBST for 2 h at room temperature. The membrane was then incubated with the primary antibody at 4 °C overnight. After washing with TBST, the membrane was incubated with the secondary antibody for 1 h at room temperature. Finally, immunoreactive bands were visualized using the ECL Plus kit (Beyotime). The bands were analyzed and normalized to anti-GAPDH levels using ImageJ 1.6 (National Institutes of Health, Bethesda, MD, USA).

### 2.10. Statistical Analysis

All data were analyzed using SPSS 23.0 (SPSS, Chicago, IL, USA). Data are expressed as mean ± standard deviation (SD). One-way analysis of variance (ANOVA) and *t*-tests were used to analyze the data. Data were considered statistically significant at *p* < 0.05 (*, *p* < 0.05; **, *p* < 0.01).

## 3. Results

### 3.1. G. parasuis Affects the Expression of miR-129a-3p in Piglets and PAM

**Figure 1:**
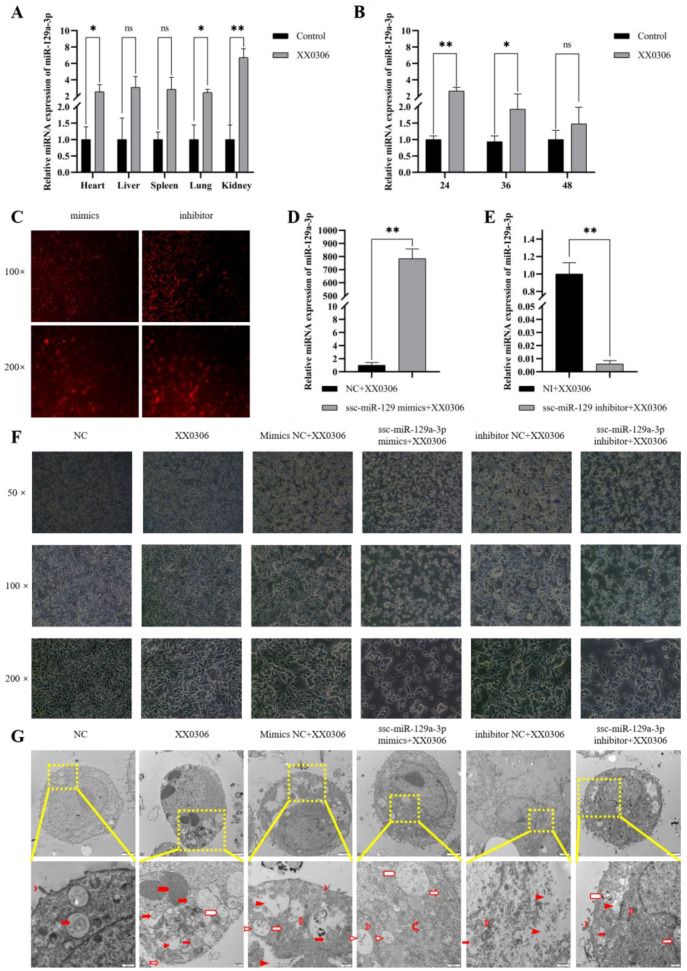
MiR-129a-3p is upregulated in different tissues and PAMs of piglets and affects the characterization of PAMs. (**A**) Expression of miR-129a-3p in different tissues of *G. parasuis*-infected piglets. (**B**) Expression of miR-129a-3p at different time points of *G. parasuis* infection with PAMs. (**C**–**E**) Transfection efficiency of miR-129a-3p detected by fluorescence microscopy and qRT-PCR. (**C**) Mimics and inhibitor are subjected to Cy3 fluorescent labeling. (**F**) Characterization of miR-129a-3p inhibitor exacerbating *G. parasuis*-infected PAMs. (**G**) Transmission electron microscopy reveals that the miR-129a-3p inhibitor exacerbates membrane damage caused by *G. parasuis* infection in PAMs. The first row displays the overall view of individual cells, while the second row shows the corresponding magnified views of each group from the first row. Different icons represent the microscopic pathological changes occurring within the cells (microvillus (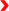); droplet (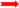); phagosome (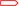); cytoplasmic lysis (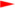); medullary structure (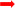); vacuole (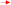); crescent (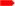); Cell membrane rupture lysis (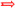); mitochondria (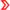); golgi apparatus (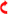); rough endoplasmic reticulum (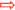); multivesicular body (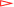); scale (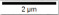)). ns *p* > 0.05 vs. control; * *p* < 0.05 vs. control; ** *p* < 0.01 vs. control.

To investigate the expression of miR-129a-3p in various organs of piglets infected with *G. parasuis*, we designed and synthesized specific primers for miR-129a-3p and conducted detection assays. Compared to the control group, the expression of miR-129a-3p significantly increased in the heart, lungs, and kidneys of *G. parasuis*-infected piglets (*p* < 0.01), while also showing an increase in the liver and spleen, though this did not reach statistical significance (Figure 1A). To further explore the expression of miR-129a-3p at the cellular level, we performed experiments on porcine alveolar macrophages (PAMs) infected with *G. parasuis*. The results showed that miR-129a-3p expression significantly increased at 24 h (*p* < 0.01) and 36 h (*p* < 0.05) after infection, reflecting the trend observed in the tissue. Additionally, over time, the expression of miR-129a-3p exhibited a time-dependent downward trend (Figure 1B). In summary, our findings reveal that the expression of miR-129a-3p increased to varying degrees across different organs of piglets and PAMs infected with *G. parasuis*.

### 3.2. miR-129a-3p Affects the Morphology of G. parasuis-Infected PAMs

To investigate the role of miR-129a-3p in PAMs, we designed and synthesized miR-129a-3p mimics and inhibitors, followed by experimental transfection. In comparison to the control group, the expression of miR-129a-3p was significantly increased in the transfected miR-129a-3p mimic group, while it was significantly decreased in the transfected miR-129a-3p inhibitor group (Figure 1C–E) (*p* < 0.01).

To assess the impact of miR-129a-3p on the morphology of *G. parasuis*-infected PAMs, we utilized an inverted microscope to observe PAM morphology at various magnifications (Figure 1F). The results indicated that the cell count in the XX0306 group was reduced, and the morphology was altered compared to the control group. The miR-129a-3p mimics combined with XX0306 exhibited a significant reduction in cell count and a considerable number of cell deaths relative to the control group. Similarly, the miR-129a-3p inhibitor combined with XX0306 also showed a significant decrease in cell count compared to the control group. The miR-129a-3p inhibitor combined with XX0306 led to an increase in cell count and altered cell morphology when compared to the miR-129a-3p mimics combined with XX0306. In summary, our findings indicate that both miR-129a-3p mimics and inhibitors exhibit effective transfection efficiency and that miR-129a-3p influences the morphology of *G. parasuis*-infected PAMs.

### 3.3. miR-129a-3p Affects Lesion Characterization of G. parasuis-Infected PAMs

To investigate the effect of miR-129a-3p on the microscopic lesion characteristics of *G. parasuis*-infected PAMs, we used transmission electron microscopy to observe the internal structure of cells. The results showed that the cell membrane was broken and dissolved, the medullary structure was increased, and the heterochromatin in the nucleus was cohesive and bordered in a crescent shape in the XX0306 group compared with that of the control group. The inhibition of miR-129a-3p could increase microvilli on the cell surface compared with the XX0306 group and could aggravate the cell damage caused by XX0306, blur the nuclear membrane, and cause a large number of cytoplasmic cleavages compared with the miR-129a-3p mimic group (Figure 1G). The above results indicated that miR-129a-3p can alleviate the cell membrane lysis caused by *G. parasuis* in PAMs, and that cytoplasm lysis is lighter compared with the knockdown group. However, cell membrane lysis and cytoplasm lysis are often due to the inflammatory response in cells. Therefore, it is speculated that miR-129a-3p may play an important role in inflammatory response.

### 3.4. Differential Expression Analysis and Functional Annotation of mRNA After miR-129a-3p Inhibition

**Figure 2:**
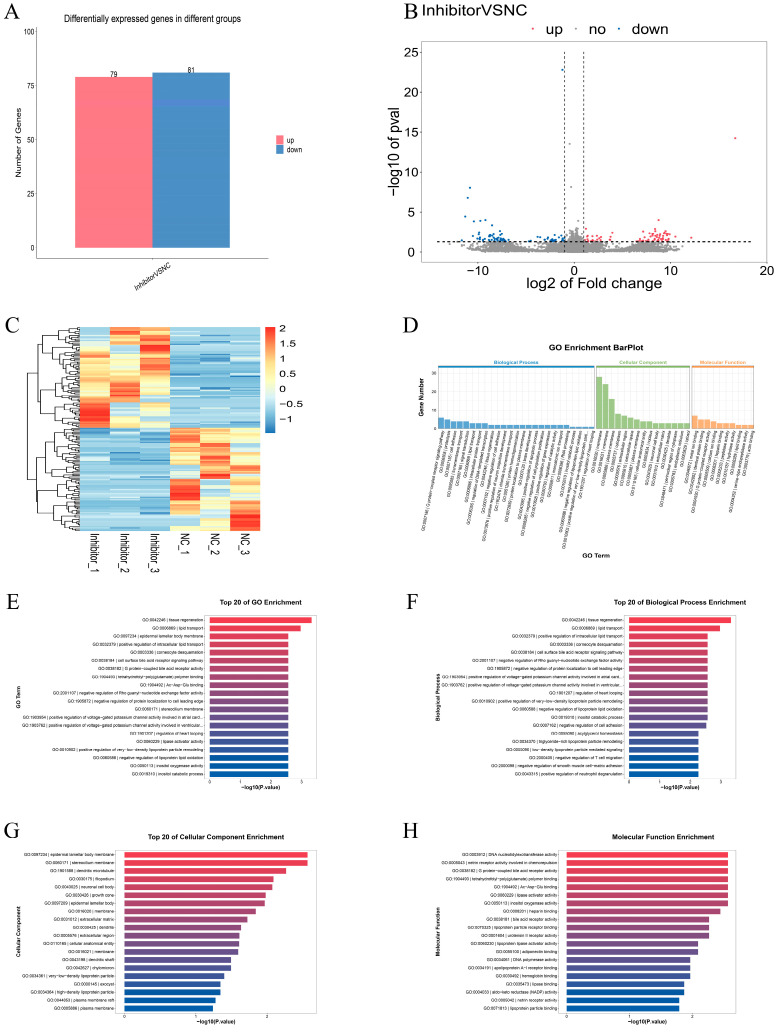

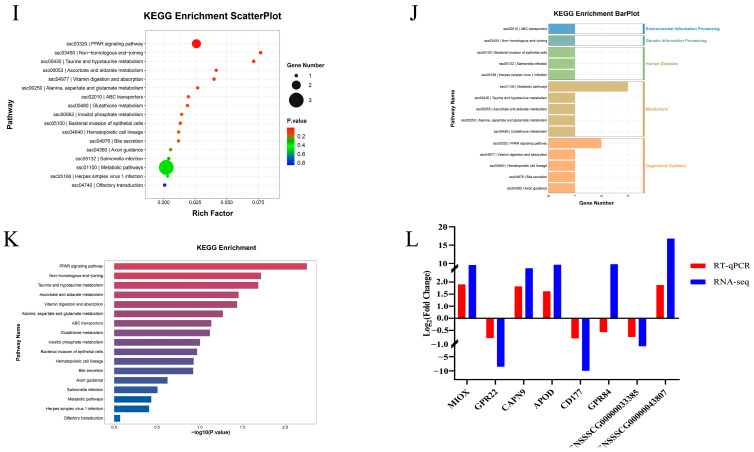
Differentially expressed genes analysis. (**A**) Differentially expressed genes visualized by histogram. (**B**) Differentially expressed genes visualized by volcano plot. (**C**) Differential gene clustering heat map. The *x*-axis of the heatmap represents the samples, while the *y*-axis represents the differentially expressed genes that have been filtered (since the total number of differentially expressed genes is large, the gene names are not displayed on the right side, only the overall trend of expression changes is shown). (**D**) The terms are sorted from large to small according to the number of annotated differential genes, and the top 25, top 15, and top 10 terms are displayed. The GO enrichment classification bar chart does not consider the enrichment significance *p*-value. The primary consideration is the number of differentially expressed genes enriched in each GO entry, sorted from largest to smallest based on the number of differentially expressed genes. (**E**) Enrichment map of the top 20 GO entries with the smallest *p* value. (**F**) Enrichment map of the top 20 GO BP entries with the smallest *p* value. (**G**) Enrichment map of the top 20 GO CC entries with the smallest *p* value. (**H**) Enrichment map of the top 20 GO MF entries with the smallest *p* value. (**I**) Bubble plot of KEGG enrichment analysis of differentially expressed genes. (**J**) Enrichment of differential gene numbers to the first-level classification map of all KEGGs. (**K**) Bar chart of KEGG differentially expressed gene enrichment analysis. (**L**) RT-qPCR validation of RNA-seq results.

To investigate the target genes and function of miR-129a-3p, we detected the differentially expressed genes after miR-129a-3p inhibition, and performed GO and KEGG pathway analysis. The results showed that compared with the NC group, 160 mRNAs were significantly differentially expressed after miR-129a-3p inhibition, including 79 up-regulated mRNAs and 81 down-regulated mRNAs (Figure 2A,B) (*p* < 0.01). Cluster analysis was performed on all differential genes (Figure 2C).

Differentially expressed genes (DEGs) were analyzed for Gene Ontology (GO) function enrichment using GO function annotation technology. DEGs were enriched in 386 GO entries, which included terms associated with the G protein-coupled receptor signaling pathway and cell adhesion (Figure 2D). Studies have shown that G protein-coupled receptors and NF-κB have a potential relationship in inflammatory responses [35]. At the same time, NF-κB is a typical speech and behavior signaling pathway [35]. Figure 2E–H show the top 20 GO entries with a significant enrichment of DEGs in BP, CC, and MF, respectively.

Additionally, KEGG enrichment analysis was performed on the DEGs. The DEGs demonstrated significant enrichment across 17 KEGG entries, with notable enrichment identified in five signaling pathways, including the PPAR signaling pathway, non-homologous end joining, and taurine and hypotaurine metabolism (Figure 2I–K). Interestingly, some studies have found that PPAR can mediate NF-κB and thereby affect the inflammatory response of cells [36].

In addition, we also verified the accuracy of eight randomly selected DEGs using the RT-qPCR method. The results showed that the expression trend of eight DEGs with sequencing was consistent with the RT-qPCR results (Figure 2L) (*p* < 0.01).

### 3.5. MiR-129a-3p Affects the Inflammatory Response to G. parasuis Infection in PAMs

**Figure 3:**
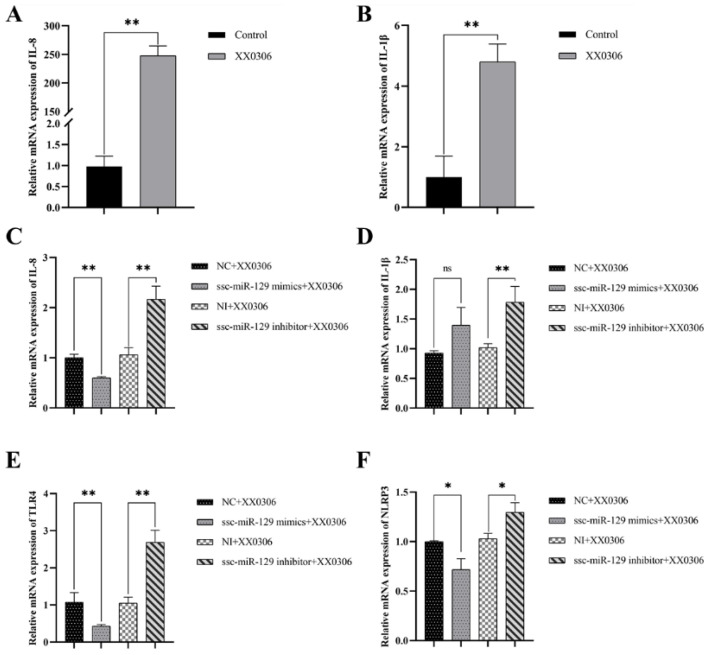
Detection of inflammatory factors and inflammatory signaling molecules. (**A**,**B**) *G. parasuis* promotes the expression of IL-8 and IL-1β in PAM. (**C**,**D**) Knockdown of miR-129a-3p promotes the expression of IL-8 and IL-1β in *G. parasuis*-infected PAMs. (**E**,**F**) Knockdown of miR-129a-3p promotes the expression of TLR4 and NLRP3 in *G. parasuis*-infected PAMs. ns *p* > 0.05 vs. control; * *p* < 0.05 vs. control; ** *p* < 0.01 vs. control.

Inflammatory factors serve as crucial indicators of cellular inflammation. IL-8 and IL-1β can be stimulated and produced in large quantities by LPS, which is an important component of *G. parasuis*. So, we investigated the alterations in the levels of inflammatory factors in *G. parasuis*-infected PAMs and observed that the expression of IL-8 and IL-1β was significantly elevated in PAMs (Figure 3A,B) (*p* < 0.01). Notably, IL-8 expression was significantly reduced in cells after overexpressing miR-129a-3p. Conversely, the inhibition of miR-129a-3p led to a significant increase in IL-8 and IL-1β levels (Figure 3C,D) (*p* < 0.01). In conclusion, miR-129a-3p plays a significant role in the inflammation response in *G. parasuis*-infected PAMs.

### 3.6. MiR-129a-3p Directly Targets the 3′-UTR of TLR4 and NLRP3

**Figure 4:**
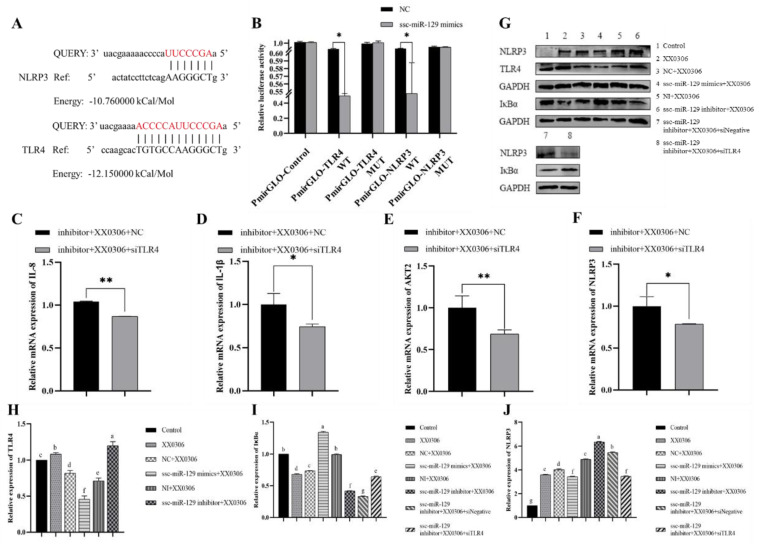
MiR-129a-3p regulates the inflammatory response of *G. parasuis* infection in PAMs via TLR4/NFκB signaling pathway. (**A**) Target prediction results of miR-129a-3p with TLR4 and NLRP3. The red text represents the predicted binding site (**B**) Dual-luciferase assay results of miR-129a-3p mimics co-transfected with recombinant vector. (**C**–**F**) Changes in intracellular IL-8, IL-1β, AKT2, and NLRP3 after co-transfection of ssc-miR-129a-3p inhibitor with siTLR4. (**G**) Western blot results of TLR4, NLRP3, and IκBα protein expression. (**H**) Quantification of TLR4 protein expression. (**I**) Quantification of IκBα protein expression. (**J**) Quantification of NLRP3 protein expression. The same letters in this figure indicate no significant difference (*p* > 0.05), while different letters indicate a significant difference (*p* < 0.05). * *p* < 0.05 vs. control; ** *p* < 0.01 vs. control.

Based on transcriptome sequencing prediction and online base complementation prediction, we discovered that TLR4 and NLRP3, two key regulators of inflammatory response, have binding sites on miR-129a-3p (Figure 4A). And, we observed that miR-129a-3p can regulate the expression levels of TLR4 and NLRP3 (Figure 3E,F) (*p* < 0.01). Interestingly, their expression trend is consistent with that of inflammatory factors. Consequently, we hypothesized that TLR4 and NLRP3 may serve as target genes for miR-129a-3p.

To validate that TLR4 and NLRP3 are direct targets of miR-129a-3p, we constructed PmirGLO-TLR4 3′-UTR and PmirGLO-NLRP3 3′-UTR recombinant plasmids and conducted luciferase reporter gene assays. The results indicated that the overexpression of miR-129a-3p significantly diminished the expression activity of the dual-luciferase reporter genes when TLR4 3′-UTR and NLRP3 3′-UTR were in their wild-type forms (Figure 4B) (*p* < 0.05). In contrast, miR-129a-3p did not influence the expression of the dual-luciferase reporter gene when TLR4 3′-UTR and NLRP3 3′-UTR were mutated. Collectively, these findings suggest that miR-129a-3p directly targets porcine TLR4 3′-UTR and NLRP3 3′-UTR.

### 3.7. TLR4 siRNA Partially Reverses Changes in miR-129a-3p Inhibitor Associated with Inflammatory Factors and Inflammatory Signaling Pathway Molecules in G. parasuis-Infected PAMs

In a previous study, the miR-129a-3p inhibitor significantly elevated the levels of inflammatory factors and molecules associated with inflammatory signaling pathways compared to the control (*p* < 0.05). To investigate the role of TLR4 in the infection of PAMs by *G. parasuis*, we designed TLR4 siRNA. However, the knockdown of TLR4 can reverse the expression of inflammatory factors after miRNA inhibition (Figure 4C–F) (*p* < 0.05). Therefore, we speculate that the miR-129a-3p inhibitor inhibits the expression of intracellular miR-129a-3p and thereby increases the expression of inflammatory factors IL-8 and IL-1β and the inflammatory signaling pathway molecule NLRP3, while the addition of siRNA-TLR4 offsets the overexpression of these molecules, leading to altered levels of inflammation. Collectively, the results of the present study suggest that miR-129a-3p regulates inflammation in PAMs to some extent by targeting TLR4.

### 3.8. MiR-129a-3p Regulates Inflammation Through TLR4/NF-κB in G. parasuis-Infected PAM Cells

To investigate whether miR-129a-3p further participates in regulating the NF-κB pathway, downstream of TLR4, during *G. parasuis* infection, we detected the key proteins of NF-κB via Western blot. The results showed that *G. parasuis* significantly decreased the expression of IκBα, thus activating the NF-κB pathway (*p* < 0.05). MiR-129a-3p significantly increased the expression of IκBα (*p* < 0.05), while inhibiting miR-129a-3p decreased the expression of IκBα (*p* < 0.05), indicating that miR-129a-3p could be involved in the regulation of IκBα. After the overexpression of miR-129a-3p, the protein expression levels of TLR4 and NLRP3 were significantly lower than those in the control group, which was consistent with the mRNA level trend (*p* < 0.05). The co-transfection of miR-129a-3p and si-TLR4 showed a significant decrease in the expression of NLRP3 (*p* < 0.05), and a significant increase in the expression of IκBα (*p* < 0.05) compared with the control group. These findings indicate that miR-129a-3p modulates inflammation via the TLR4/NF-κB pathway in *G. parasuis*-infected PAM cells.

## 4. Discussion

*G. parasuis* is a significant pathogenic bacterium responsible for Glässer’s disease, which incurs substantial economic losses to the pig industry [37]. Recent studies have indicated that miRNAs are crucial in the pathogenesis of various diseases, including Huntington’s disease, Alport syndrome, and wounds [38]. Notably, some miRNAs are implicated in diseases caused by *G. parasuis*; for example, the miRNA-FOXO1/4 axis participates in regulating skeletal muscle atrophy caused by *G. parasuis* in piglets [39]. In the present study, we observed that the expression of miR-129a-3p was upregulated to some extent in various organs following *G. parasuis* infection in piglets. Consequently, we hypothesize that miR-129a-3p may play a role during the infection period of *G. parasuis*.

It has been reported that the morphology and pathology of *G. parasuis*-infested PAM cells are altered [40]. Similarly, in this study, inverted microscopy and transmission electron microscopy were used to observe that the cell membrane was damaged and dissolved, and heterochromatin in the nucleus condensed and edged into a crescent shape, which was consistent with previous findings. More importantly, we discovered that miR-129a-3p could influence the morphological and pathological changes in *G. parasuis*-infected PAM cells. It is worth noting that, at the microscopic level, the knockdown of miR-129a-3p further aggravated the lysis of *G. parasuis*-infected PAM cytoplasm and increased the number of phagosomes, and these phenomena are mostly caused by inflammation, indicating that miR-129a-3p may play a key role in mediating the inflammatory response of *G. parasuis*-infected PAM cells. In this study, the RNA-seq technique was employed to analyze the effects of knockdown versus non-knockdown miR-129a-3p in *G. parasuis*-infected PAM cells. A total of 160 differentially expressed genes (DEGs) were identified in the miR-129a-3p inhibitor group. Gene Ontology (GO) enrichment analysis was conducted on these DEGs, revealing a total of 386 enriched GO entries, including pathways related to G protein-coupled receptor signaling and cell adhesion. It has been reported that G protein-coupled receptors play a significant role in inflammatory pathways [35]. For instance, TGR5 acts as a negative regulator of gastritis by antagonizing the NF-κB signaling pathway, suggesting that TGR5 mitigates gastritis, at least in part, by inhibiting NF-κB signaling [41]. Additionally, GRK2/β-arrestin2 has been implicated in various inflammatory diseases [42]. Furthermore, GPR84 signaling is critical for promoting pro-inflammatory myeloid responses during the early stages of acute wound healing [43]. To further elucidate the impact of DEGs in *G. parasuis*-infested PAM cells, KEGG enrichment analysis was performed on the identified DEGs from the RNA-seq data. The results indicated that DEGs were significantly enriched in 17 KEGG pathways, particularly the PPAR signaling pathway (*p* < 0.05). PPAR (peroxisome proliferator-activated receptor) is a class of nuclear receptor involved in regulating inflammatory responses and immune reactions. These findings further underscore the role of miR-129a-3p in the inflammatory damage associated with *G. parasuis*-infected PAM cells.

**Figure 5:**
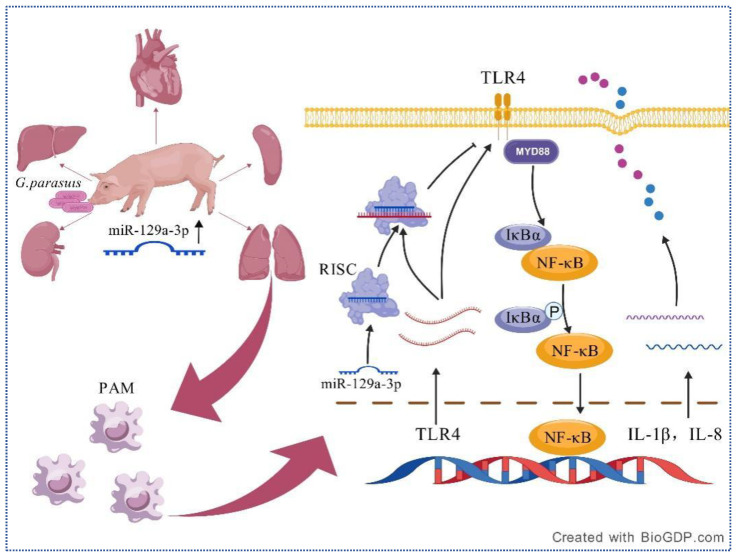
A summary schematic diagram of the mechanism by which miR-129a-3p negatively regulates the inflammatory response to *G. parasuis*-infested PAMs by targeting the TLR4/NF-κB signaling pathway.

MicroRNAs (miRNAs) regulate gene expression by binding to the 3′ untranslated region (3′ UTR) of target mRNAs, leading to mRNA cleavage or translational repression [44]. Toll-like receptor 4 (TLR4) serves as a crucial inflammatory marker; for instance, during bacterial infection, TLR4 can induce inflammation [45]. The diathesis resulting from the sustained propagation of TLR4-mediated inflammation contributes to further ischemic damage and neurocognitive deficits [46]. TLR4 also modulates Helicobacter pylori-induced inflammatory responses [47]. Recent studies have found that Han-miR3630-5p in GENs can bind to the 3′ untranslated region of TLR4, thereby inhibiting the expression of TLR4. The intake of GENs significantly downregulates the expression of TLR4, MyD88, and NF-κB, thereby suppressing the downstream cascade reaction and leading to a reduction in the secretion of DSS-induced pro-inflammatory cytokines. Similarly, in a study investigating the effects of PPF on intestinal mucosal permeability and bacterial invasion, it was found that miR-155 in mouse macrophages regulates the secretion of inflammatory cytokines through the TLR4/NF-κB pathway [48]. In a study examining the improvement of sepsis-induced lung injury by Shikonin through the modulation of miRNA-140-5p/TLR4-a in vitro and in vivo, it was discovered that miRNA-140-5p targets the TLR4 gene, leading to the suppression of TLR4 expression and the inhibition of downstream MyD88 and NF-κB protein expression [49]. A recent study on type 2 cardiorenal syndrome (CRS) revealed that ZWD could upregulate the expression of miR-451 in renal tissues, inhibit the TLR4/NF-κB/HIF-1α response loop, and subsequently suppress inflammatory factors [50]. The NLRP3 inflammasome plays a significant role in the inflammatory response, and studies identified NLRP3 as a key contributor to the formation of inflammation [51,52]. Notably, palmitoylation may prevent sustained inflammation by limiting NLRP3 inflammasome activation through chaperone-mediated autophagy [45]. In the current study, TLR4 and NLRP3 were found to be upregulated in primary alveolar macrophages (PAMs) with the knockdown of miR-129a-3p, further confirming that they are direct targets of miR-129a-3p. Both TLR4 and NLRP3 are signature molecules involved in the genesis of inflammation [53,54]. Therefore, the inflammation triggered by *G. parasuis*-infected cells may be associated with a potential link between miR-129a-3p and the inflammatory pathway. Additionally, IκBα has been implicated as a major regulator of the NF-κB signaling pathway [55]. The silencing of IκBα has been shown to activate inflammation by enhancing NF-κB’s entry into the nucleus, whereas TLR4 expression inhibits IκBα [55,56,57]. The above findings indicate that multiple miRNAs can target TLR4 and thereby regulate the NF-κB signaling pathway. This is similar to the TLR4 inhibition mediated by miR-129a-3p observed in this study, which aligns with known NF-κB regulators. This study observed the effect of miR-129a-3p on the NF-κB signaling pathway in *G. parasuis*-infected PAM cells, demonstrating that miR-129a-3p can inhibit the activation of the NF-κB signaling pathway and modulate *G. parasuis*’s response to inflammatory factors. This modulation occurs through the downregulation of TLR4 and the subsequent elevation of IκBα protein expression, thereby influencing the inflammatory responses associated with *G. parasuis* (Figure 5). The miR-129a-3p identified in this study provides a novel miRNA regulator for the TLR4/NF-κB signaling pathway.

Glässer’s disease is widely prevalent in the global pig farming industry, imposing a significant economic burden on the sector. Currently, there are more than 15 serotypes of *G. parasuis*, with poor cross-protection between different serotypes, making it difficult for a single vaccine to cover all prevalent strains. This adds to the challenges in controlling Glässer’s disease. In addition to traditional inactivated vaccines or subunit vaccines, new types of vaccines are gradually entering the market, such as miRNA vaccines. In this study, it was found that miR-129a-3p regulates the inflammatory injury of PAM cells infected with Streptococcus suis through the TLR4/NF-κB signaling pathway, highlighting the significant role of miR-129a-3p. Therefore, miR-129a-3p can be considered as a candidate target for vaccines. Additionally, further research can be conducted on the use of miRNA as a vaccine adjuvant, such as investigating miR-129a-3p as a vaccine adjuvant to enhance the immune response of traditional vaccines in the body. As mentioned in the previous discussion, miRNA can serve as both a diagnostic marker and a therapeutic tool. Therefore, miR-129a-3p in this study provides a new reference for miRNA diagnostic and therapeutic markers, offering novel insights for enhancing the prevention and control of Glässer’s disease in practical production.

## 5. Conclusions

MiR-129a-3p negatively regulates the inflammatory response to *G. parasuis* in PAMs through the miR-129a-3p/TLR4/NF-κB signaling pathway. These results provide a basis for exploring the mechanism of Glässer inflammation. However, further studies are needed to determine the role of miR-129a-3p in vivo and should focus on elucidating further potential mechanisms and conducting clinical trials. MiR-129a-3p provides a new reference as a vaccine candidate adjuvant and diagnostic marker, offering novel insights for enhancing the prevention and control of Glässer’s disease in practical production.

## Figures and Tables

**Table 1:** Sequences for overexpression and interference.

Names	Sequence (5′-3′)
mimic NC	sense: UUCUCCGAACGUGUCACGUTT
	antisense: ACGUGACACGUUCGGAGAATT
inhibitor NC	sense: CAGUACUUUUGUGUAGUACAA
ssc-miR-129a-3p mimics	sense: AAGCCCUUACCCCAAAAAGCAU
	antisense: GCUUUUUGGGGUAAGGGCUUUU
ssc-miR-129a-3p inhibitor	sense: AUGCUUUUUGGGGUAAGGGCUU
NC	sense: UUCUCCGAACGUGUCACGUTT
siTLR4	sense: CAGGAAUCCUGGUCUAUAATT

**Table 2:** Primer sequences for miRNA quantitative experiment.

Gene Name	Accession No.	Primer Sequence (5′-3′)
ssc-miR-129a-3p	MIMAT0013959	RT: GTCGTATCCAGTGCAGGGTCCGAG GTATTCGCACTGGATACGACATGCTT
		F: CGAAGCCCTTACCCCAAA
		R: AGTGCAGGGTCCGAGGTATT
U6	XM_021101534.1	RT: AACGCTTCACGAATTTGCGT
		F: CTCGCTTCGGCAGCACA
		R: AACGCTTCACGAATTTGCGT

**Table 3:** Primer sequences for genetic testing.

Gene Name	Accession No.	Primer Sequence (5′-3′)	Product Size (bp)
GAPDH	NM_001206359.1	F: CACAGTCAAGGCGGAGAAC	106
		R: CGTAGCACCAGCATCACC	
IL-1β	XM_021085847.1	F: TGATGCCAACGTGCAGTCTA	92
		R: GGAGAGCCTTCAGCATGTGT	
IL-8	NM_213867.1	F: TCCAAACTGGCTGTTGCCTTCTTG	132
		R: GGGGTGGAAAGGTGTGGAATGC	
AKT2	XM_013988560.2	F: AAAGTCATCCTGGTGCG	137
		R: GGGTGCCTGGTGTTCTG	
NLRP3	NM_001256770.2	F: GGAGGAGGAGGAAGAGGAGATA	147
		R: AGGACTGAGAAGATGCCACTAC	
TLR4	NM_001113039.2	F: CGTCAGTTCTCACCTTCCTCC	165
		R: CATTCCTCACCCAGTCTTCGT	

**Table 4:** Primer sequences for verification.

Gene Name	Accession No.	Primer Sequence (5′-3′)
ENSSSCG00000033385	ENSSSCG00000033385	F: GCACGCTTAGGGAGGAACAA
		R: AGGACCTCTGGTCGGTAGTC
ENSSSCG00000043807	ENSSSCG00000043807	F: TGGGAATGTTTGGACCTTCGT
		R: TTTTGCTCCAACAAGGGAACC
MIOX	ENSSSCG00000023749	F: CTCTCAGGATGAAGGACCCAG
		R: AGCTTGTAGGTGCGGAAGAC
GPR22	ENSSSCG00000015442	F: CTGAGTCTCTTCTCCCAGTCCT
		R: ACTTGCCAGTTCAAAAGCAGC
CAPN9	ENSSSCG00000010182	F: AGAATGCGAGCCGGATGTTC
		R: CAACCAAGACTCCTCGGGAC
APOD	ENSSSCG00000011831	F: ATCTGAGCACGTTTGTCCCA
		R: GCCTAAAAGCTGAGCTCGTG
GPR84	ENSSSCG00000000291	F: AAGGCCTAGATTTTGGAGTGGA
		R: GCCATTCCCAGGCCTCTTTA
CD177	ENSSSCG00000003051	F: ATGGACCACAAGTGCGGAG
		R: AGGAGTGATCTGTGTCCTGC

**Table 5:** Plasmid sequences used for dual-luciferase detection.

Gene Name	Sequence (5′-3′)	Size (bp)
TLR4 WT	CATGATACAACAGCCTTCACTTAAGGAGGGAAAACTCCCAACGTGTCCCTTGGTCAGCTGGATCCCGTGCTTGTTAACAAGTACTAAATCCTGCAACATGCCAAGCACTGTGCCAAGGGCTGGTGATTCAGTGATGCCCGAGATACACAGGACTGCCAGTCTCGTGGAGTTTACAATTTAGAGGGACTAAACACTGTTCTAAAATACAGAACTTCCAGGTGG	222
TLR4 MUT	CATGATACAACAGCCTTCACTTAAGGAGGGAAAACTCCCAACGTGTCCCTTGGTCAGCTGGATCCCGTGCTTGTTAACAAGTACTAAATCCTGCAACATGCCAAGCACACTCCCTTCCCGAGGTGATTCAGTGATGCCCGAGATACACAGGACTGCCAGTCTCGTGGAGTTTACAATTTAGAGGGACTAAACACTGTTCTAAAATACAGAACTTCCAGGTGG	222
NLRP3 WT	GCAAGCTAAAGAAGCTCTGGTTGGTCAGTTGCTGTCTCACATCAGCGTGTTGTGAGGATCTTGCGTCCGTCCTGAGCAGCAATCATTCCCTGACCAGACTATAGACCCCAAGTTAAGGGCTCATGCCCTGGGAGACTCAGGAGTTGGAATTTTATGTGAAAAAGCAAAGCATCCACAATGTAACCTGCAAAAACTGGGGTTGGTGAATTCTGGCCTTACATC	222
NLRP3 MUT	GCAAGCTAAAGAAGCTCTGGTTGGTCAGTTGCTGTCTCACATCAGCGTGTTGTGAGGATCTTGCGTCCGTCCTGAGCAGCAATCATTCCCTGACCAGACTATAGACCCCAACTATTCCCGACATGCCCTGGGAGACTCAGGAGTTGGAATTTTATGTGAAAAAGCAAAGCATCCACAATGTAACCTGCAAAAACTGGGGTTGGTGAATTCTGGCCTTACATC	222

**Table 6:** The antibodies used in the present study.

Names	Dilution Rate	Manufacturer	Product Number
GAPDH	1:10,000	Proteintech, Wuhan, China	10494-1-AP
IkB Alpha	1:2000	Proteintech, Wuhan, China	10268-1-AP
NLRP3	1:300	Proteintech, Wuhan, China	19771-1-AP
TLR4	1:1000	ABclonal, Wuhan, China	A5258

## Data Availability

Data will be made available upon reasonable request.
